# Impact of multi-step vs one-step finishing and polishing techniques on color stability of omnichroma composite restorations: In vitro spectrophotometric analysis

**DOI:** 10.4317/jced.64171

**Published:** 2026-06-29

**Authors:** Giorgia Lanzaretti, Khalil Fari, Thiago Oliveira-Gamba, Francesca Zotti

**Affiliations:** 1 DDS. Private practice, Vicenza, Italy; 2 DDS. Private practice, Trento, Italy; 3 Adjunt Professor. School of dentistry, Federal university of Rio Grande do Sul (UFRGS), Porto Alegre, Rio Grande do Sul, Brazil; 4 Associate professor, PhD. Department of Surgical Sciences, Paediatrics and Gynaecology, University of Verona, P.le L.A.Scuro, 10, 37134 Verona, Italy

## Abstract

**Background:**

This in vitro study aimed to analyze, using a spectrophotometer, the outcomes of two finishing and polishing techniques for composite restorations and to evaluate their color stability after exposure to a staining agent.

**Materials and Methods:**

Standardized class V cavities were prepared on sixty extracted intact molars and restored with Omnichroma composite (Omnichroma, Tokuyama Dental Corporation Inc., Tokyo, Japan) . The samples were divided into two groups: Group 1, finished and polished with the multi-step Astropol technique (Ivoclar Vivadent, Liechtenstein), and Group 2, finished and polished with the one-step Enhance technique (Dentsply Sirona, USA). Color changes were evaluated using a spectrophotometer immediately after polishing with each technique (T1) and after two weeks of immersion in a staining agent (T2). Color differences (E) were calculated for intergroup and intragroup analyses.

**Results:**

No statistically significant differences were detected immediately after polishing (T1, p=0.059); however, significant variations emerged after immersion in the staining agent (T2, p&lt;0.001).

**Conclusions:**

Intragroup analysis showed significant E changes for the Enhance group (p&lt;0.001) but not for the Astropol group (p=0.724). Both groups, however, exhibited E &gt; 2, indicating a perceptible color difference between the restoration and sound dental tissue.

## Introduction

The continuous pursuit of aesthetic excellence in restorative dentistry has led to the development of innovative materials, among which single-shade composite resins represent a significant advancement. These materials, such as Omnichroma (Tokuyama Dental Corporation Inc., Tokyo, Japan), are designed to adapt to the color of the surrounding tooth structure through complex optical phenomena, reducing the need for multiple shades and simplifying the restorative procedure. While their immediate color blending capabilities are well-documented, less is understood about how different finishing and polishing techniques influence their long-term color stability and chromatic integration, particularly given their unique optical properties. The finishing and polishing phases are critical for optimizing the aesthetic outcome and longevity of composite restorations. A smooth, glossy surface not only enhances the immediate aesthetics but also minimizes bacterial plaque accumulation, reduces the risk of secondary caries and gingival inflammation, and crucially, helps prevent extrinsic staining and discoloration over time. Therefore, understanding the interplay between advanced composite materials and various finishing/polishing protocols is essential for achieving predictable and durable aesthetic results. In direct restorations, the literature reports that a significant percentage of failures are aesthetic, most of all in anterior teeth; as patients often complain about an imperfect integration of the restoration with the dental structures (1 , 2). This could be due to wrong color choice, improper shape or irregularities in the composite surface that hinder a correct adaptation of the restoration to sound dental tissue. A smooth and well-polished surface allows for better integration of the restoration with the surrounding dental tissue and prevents discoloration, but also prevents bacterial plaque accumulation, reducing the risk of caries and gum disease (3). Finishing is one of the final processes of a dental restoration, involving correction of shape irregularities and removal of any excess material, while polishing is performed after the finishing phase with the aim of creating a homogeneous, smooth, and glossy surface, improving the aesthetic properties of composite resins. The finishing and polishing process involves the use of a sequence of abrasive tools and materials on the restoration surface that are made of diamond, silicon carbide or aluminium oxide, which have a hardness greater than that of the restoration surface. The instruments sequence usually starts with a coarser abrasive material that removes macroscopic surface irregularities and then proceeds to abrasives with finer grit to obtain a smoother and shinier surface (4). The color of the dental restorative material used must be maintained throughout its functional lifespan to ensure that the aesthetic quality of the restoration remains satisfactory. Color changes in resin material, and thus their discoloration, though, can be intrinsic or extrinsic. Intrinsic discoloration is permanent and is related to the composition of the restorative material, including matrix, type and quantity of the filler, type of photo initiators, and percentage of remaining carbon double bonds (5 , 6). These chemical and physical reactions are located within the deeper layers of the composite resin. Extrinsic discoloration, on the other hand, may result from a plaque accumulation and related stains, reduced polymerization, or exposure to environmental factors such as heat, water, staining agents (e.g., beverages, smoking, mouthwash, etc.), ambient light, and ultraviolet rays. Although discoloration is often multifactorial, extrinsic factors appear to exert a more pronounced effect on long-term color stability and aesthetics in composite restorations. Color adaption in restorative dentistry is commonly assessed through the calcunation of E between two spectral images, mathematically expressed as follows: E = [(L2 L1)² + (a2 a1)² + (b2 b1)²] Color differences in dentistry are commonly quantified using the CIELab color space, defined by three coordinates: L* (brightness), a* (red-green chroma), and b* (yellow-blue chroma). The resultant color difference, Eab*, is calculated using the Euclidean distance formula. While Eab* has been widely adopted due to its simplicity, it is known to have limitations in correlating with visual perception, particularly in certain color region. Smaller E values indicate greater similarity between the restoration and surrounding dental tissue as perceived by the human eye. Although recent studies have investigated the staining behavior, bleaching response, polishability, and optical stability of universal or single-shade composites using standardized composite specimens, evidence remains limited regarding their chromatic performance when placed in clinically relevant cavity configurations (7). Flat composite specimens are useful for isolating material-related phenomena, such as pigment uptake, surface permeability, and response to bleaching agents; however, they do not reproduce the optical complexity of a clinical restoration, where color blending depends on the interaction between the composite, cavity geometry, adhesive interface, restoration margins, light reflection, and surrounding dental tissues. Therefore, the novelty of the present study lies in evaluating Omnichroma restorations placed in standardized Class V cavities on extracted molars, rather than in composite discs or blocks alone. This model allowed assessment of whether different finishing and polishing protocols influence not only surface-related discoloration, but also the preservation of chromatic integration between a structural-color single-shade composite and natural tooth tissue after exposure to staining. From this perspective, the study addresses a clinically relevant question: whether the simplification offered by single-shade composites can be extended to the finishing and polishing phase, or whether a multi-step polishing protocol remains necessary to maintain long-term aesthetic stability. The aims of this in vitro study were: 1. To evaluate, through spectrophotometry, the color performance achieved by two finishing/polishing techniques in Class V restorations performed with Omnichroma composite (Tokuyama Dental Corporation Inc., Tokyo, Japan); 2. To assess their color performance over time after immersion in a staining agent.

## Materials and Methods

**Figure 1:**
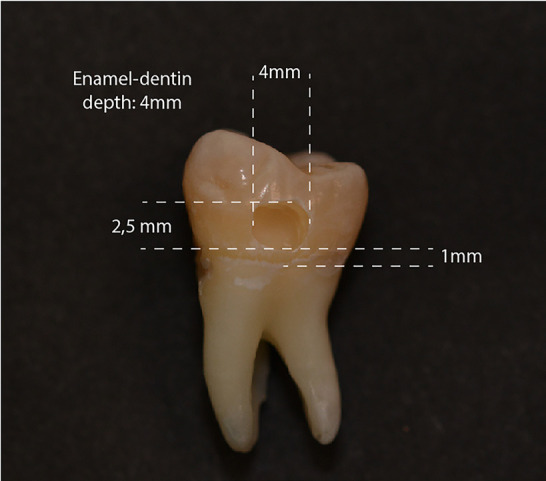
Standardized class V cavity with precise measurements in mm.

The study was approved by the Ethical Committee of South Est Veneto (approval number: 430CET). Sample size was determined using G-Power v. 3.1 statistical software (University of Düsseldorf; Düsseldorf, Germany) (8). A statistical significance analysis revealed that a sample size of 60 met the constraints of = 0.05 and power = 0.95. Sixty intact upper and lower molars free from buccal carious lesions, crown fractures, restorations, or significant abrasions/erosions were collected. Teeth presenting cracks, severe root resorption, or active periodontal disease were excluded. All specimens were cleaned of residual periodontal ligament using a curette, rinsed with denatured alcohol, washed under running water for 60 seconds, and stored in saline solution at room temperature to prevent dehydration. Informed consent for the use of extracted teeth for research purposes was obtained from all patients prior to extraction. All surgical procedures were performed as part of a treatment plan. A standardized class V cavity was prepared on the buccal surface of every dental element, under water irrigation and using a cylindrical diamond bur (1mm diameter, 5mm length) as follows: (Fig. 1).

1

**Figure 2:**
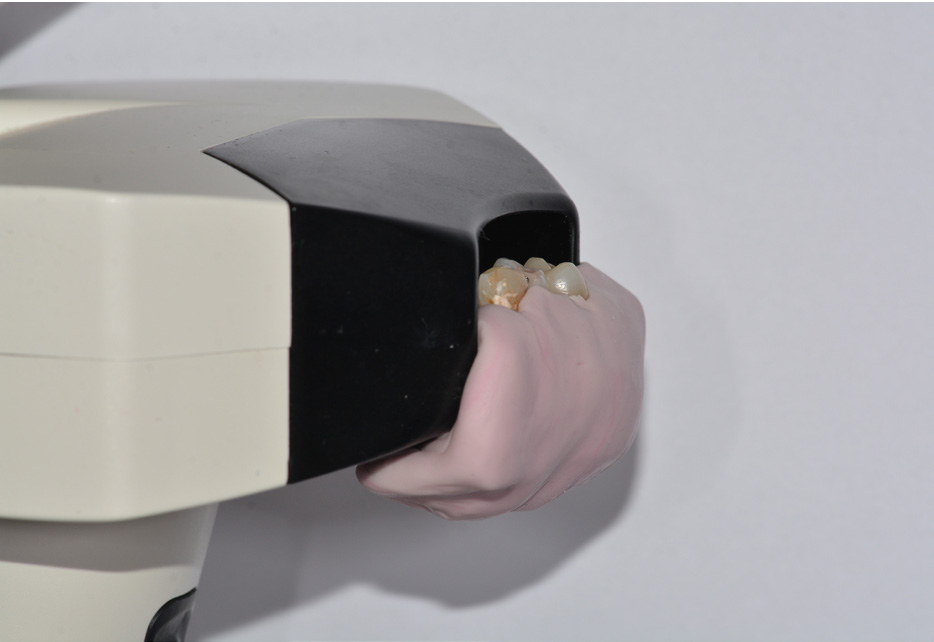
Silicone support moulded to adapt to the spectrophotometer sensor and to lodge three dental elements.

- Cervical margin: 1mm above the CEJ - Enamelo-dentin depth: 4mm - Mesio-distal width: 4mm - Corono-apical height: 2.5mm All measurements were verified with a periodontal probe, and a rubber stop was used to standardize cavity depth. All elements were restored with a standardized protocol, as follows: 1. Selective etching with Tokuyama Etching Gel HV (Tokuyama Dental Corporation Inc., Tokyo, Japan): 30 seconds on enamel and 15 seconds on dentin; 2. Acid aspiration; 3. Rinsing for 30 seconds with water; 4. Gentle air drying; 5. Adhesive procedure with Tokuyama EE Bond (Tokuyama Dental Corporation Inc., Tokyo, Japan): 10-second application on the entire cavity surface, followed by air drying; 6. Polymerization for 20 seconds; 7. Restoration using incremental layering of Omnichroma composite (Tokuyama Dental Corporation Inc., Tokyo, Japan), with 20-second polymerization time for each increment. The elements were then randomly divided into two groups of 30 using Excel (version 18.0, Microsoft Office 2021). Elements of group 1 were finished and polished using the following protocol: 1. Finishing with Astropol F cup (gray) to remove excess material and smooth the rough surfaces of the restoration. 2. Polishing with the Astropol P cup (green) to obtain a smooth surface of the restoration. 3. Polishing with the Astropol HP cup (pink) to achieve surface gloss. As indicated by the manufacturer, reduced pressure was applied in this final phase. Group 2 specimens underwent finishing using the Enhance finishing rubber cup on a low-speed handpiece. The abrasive effect of the Enhance finishing rubbers was directly influenced by the pressure exerted on the composite surface. For the purpose of this study, the Enhance finishing instrument was intentionally used as a standalone "one-step" approach to compare the effect of a simplified finishing protocol on surface smoothness and color stability against a multi-step polishing system. This protocol did not represent the complete manufacturer-recommended Enhance/PoGo polishing sequence, which includes an additional polishing step; therefore, the findings for this group should be interpreted as referring specifically to the simplified one-step protocol tested in the present study. In both groups, the finishing and polishing process was carried out using water irrigation for cooling and to remove the polishing residues. The samples were then subsequently stored in a saline solution for 24 hours. - Spectrophotometric evaluation Color differences were calculated using the Eab* formula as provided by the SpectroShadeDatabase® software (MHT SpectroShade) integrated with the spectrophotometer. All dental elements were assigned an alphanumeric code to facilitate the recording of color at different time intervals and the subsequent comparisons. For the spectrophotometric evaluation, each tooth was embedded in Putty Hard (Zetalabor, Zhermack Dental, Germany) silicone material, which was pink-coloured to simulate gingival tissue, and placed next to two additional teeth, reused for every sample evaluation (9 , 10). This precaution was necessary because the spectrophotometer can only read the color if both adjacent elements are present. The silicone support was shaped to position every specimen consistently relative to the spectrophotometer sensor, thereby standardizing measurements. (Fig. 2).

2

**Figure 3:**
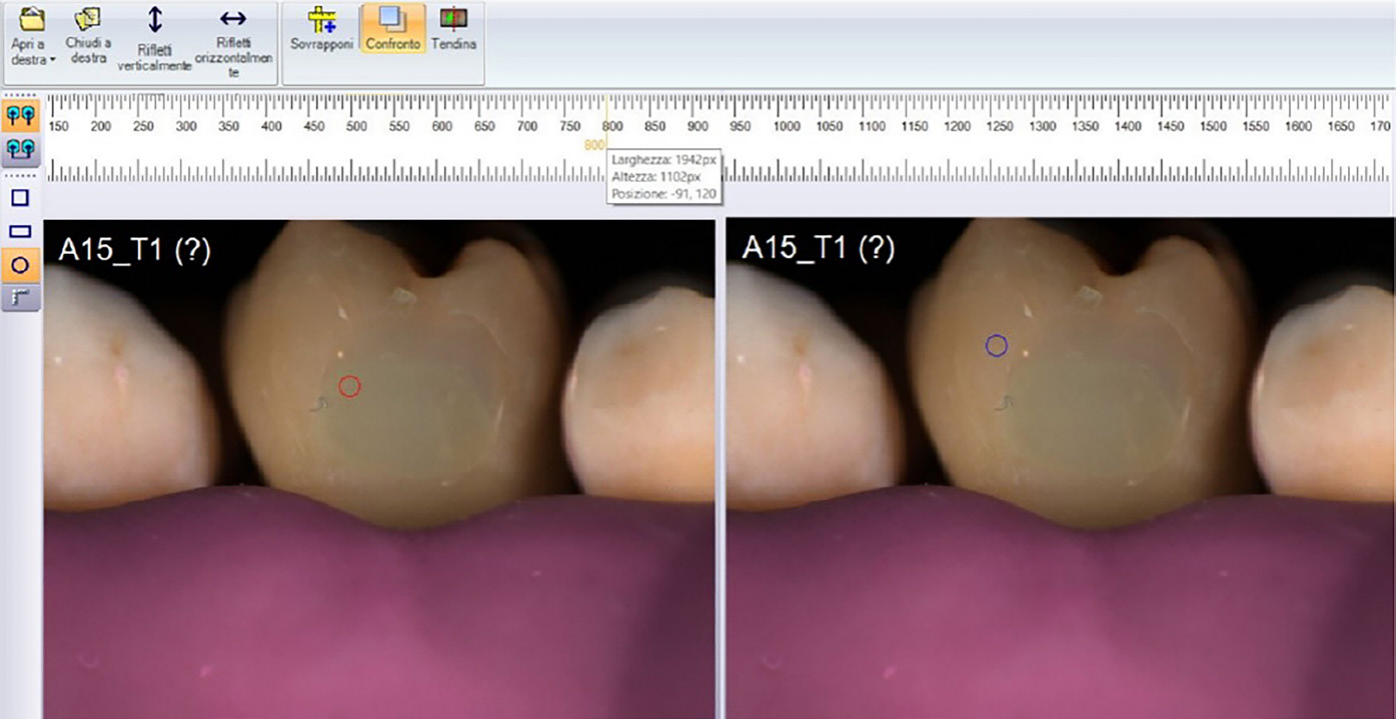
Example of use of the circular selection tool and digital ruler to standardize measurements.

To simulate the darkness of the oral cavity, a black card was placed around the sample and the sensor. Measurements were performed using a SpectroShade Micro spectrophotometer (MHT Optic Research AG, Niederhasli, Switzerland). For each measurement, a standardized circular area of 20 pixels was selected using the software's circular selection tool, ensuring consistent measurement geometry. The spectrophotometer acquires spectral data across the visible light spectrum (400-700 nm) with a resolution of 10 nm, providing precise color coordinates (L*, a*, b*) for each measured point. To ensure standardization, two coordinates (x and y) were identified and standardized for each tooth measurement using the software's tools and a digital ruler (ScreenRuler, v.0.10.0, Bluegrams). To investigate differences in color performance of the two finishing and polishing techniques (inter-group evaluation) and their behavior over time (intra-group evaluation), spectrophotometric evaluations were performed at the following time intervals: - T1: E at 24 hours after finishing/polishing with the two techniques - T2: E after 14 days of exposure to the staining agent. - Staining protocol Following the initial spectrophotometric evaluation (T1), all specimens were immersed in a staining solution for 14 days, which simulated a time exposure of 14 months (11). The staining agent consisted of freshly brewed coffee (Instant coffee, Pam Panorama S.p.a.) prepared at a ratio of 3.6 g of ground coffee per 300mL of distilled water. The coffee was dissolved in water at 90°C and cooled to room temperature before use. Each sample was individually immersed in 20mL of the prepared coffee solution in a sealed container, ensuring complete coverage. The coffee solution was changed daily to maintain consistent staining potency and prevent bacterial growth. Samples were kept at room temperature (23°C ± 1°C) throughout the immersion period. After 14 days, samples were rinsed thoroughly under running water for 60 seconds, then gently wiped dry before T2 spectrophotometric measurements. - Intra-group evaluation Intra-group evaluation aimed to assess the differences in E values between the restorations of each group at different time intervals. EAB was calculated at T1 and T2 for each dental element between point A, located inside the restoration, and point B, located on healthy dental tissue. To ensure that point A and B were always of the same size, the circular selection tool provided by the software was used and set to a size of 20 (Fig. 3).

3

Also, using the tools provided by the software and a digital ruler (ScreenRuler, v.0.10.0, Bluegrams), two coordinates (x and y) were identified, and standardized on every tooth for each point (Fig. 3). Spectrophotometer was calibrated before every measurement, according to the manufacturer's instructions and between measurements, all samples were stored in saline solution. Measurements were repeated for each dental element in both groups, resulting in the following comparisons: - Group 1 (Astropol) T1 EAB vs T2 EAB - Group 2 (Enhance) T1 EAB vs T2 EAB Inter-group evaluation Inter-group evaluation aimed to evaluate differences in E values between the restorations of the two groups, calculated at T1 and T2, resulting in the following comparisons: - E T1G1 vs E T1G2 - E T2G1 vs E T2G2 The same area was selected in both groups for each comparison to standardize measurements. Specifically, an area in the middle/cervical third was chosen, including a portion of healthy tissue and part of the restoration. Spectrophotometer was calibrated before every measurement, according to the manufacturer's instructions and between measurements, all samples were stored in saline solution. - Statistical analysis E values were collected using the spectrophotometer's software (MHT SpectroShade). Data were interpreted with STATA 16 statistical software (StataCorp, 1985, California, USA) and t tests were considered statistically significant for p 0.05. The Shapiro-Wilk statistical test was used to assess the normality of data distribution, which was found to be normal. For the intra-group evaluations, the following tests were used: - Wilcoxon-Mann-Whitney Test to assess the differences in EAB of the elements in Group 1 over the T1-T2 time interval (T1 EAB vs T2 EAB). - T-Student statistical test to assess the differences in EAB of the elements in Group 2 over the T1-T2 time interval For inter-group evaluations, E T1G1 vs E T1G2 and E T2G1 vs E T2G2, the Wilcoxon-Mann-Whitney test was used.

## Results

Tables 1 and 2 present the E values recorded 24 hours after the finishing/polishing (T1 and 14 days after immersion in the staining agent (T2) Table 3 present the descriptive statistics (mean and standard deviation) of the E values at different time intervals for both groups.

Table 1

Table 2

**Figure 4:**
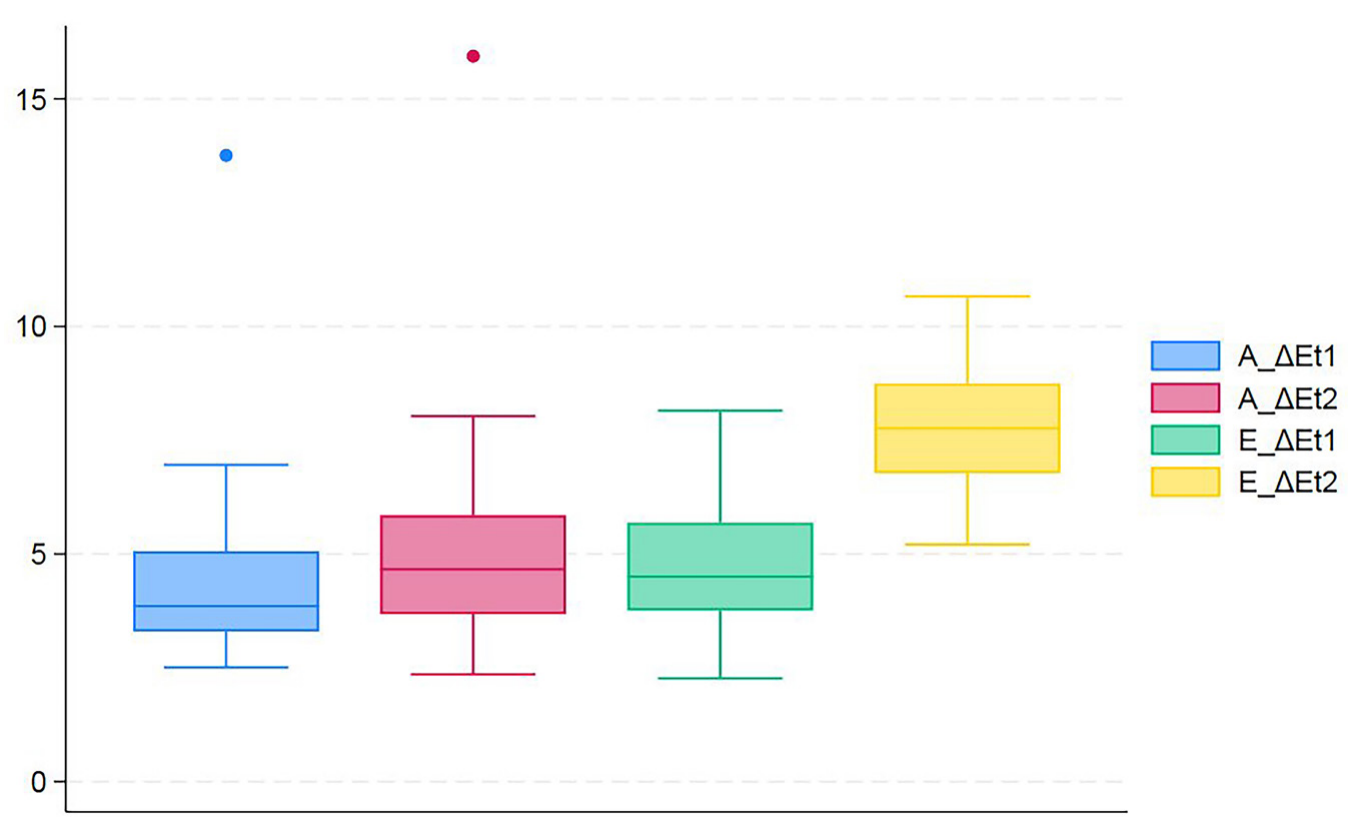
ΔEAB measurements of Groups 1 and 2 at different timepoints.

Figure 4 shows the EAB measurements of Groups 1 and 2 using a box-and-whisker plot.

4

Intra-group analysis - Group 1 (Astropol) T1 EAB vs T2 EAB The Wilcoxon-Mann-Whitney Test did not reveal statistically significant differences between the E values at T1 and T2 (p = 0.0724), indicating that there was no statistically significant change in color values before and after exposure to the staining agent for restorations finished and polished with the Astropol technique -Group 2 (Enhance) T1 EAB vs T2 EAB The T-Student Test revealed statistically significant differences between the E values at T1 and T2 (P &lt; 0.001), indicating that color values were different before and after exposure to the staining agent in restorations finished and polished with the Enhance technique. Inter-group analysis - T1 - EAB G1 vs. EAB G2 The Wilcoxon-Mann-Whitney Test did not reveal a statistically significant difference (p = 0.0594) in E values of the two groups in the first measurement, suggesting that the initial color performance of the two finishing techniques did not differ significantly after 24 hours. - T2 - EAB G1 vs. EAB G2 The Wilcoxon-Mann-Whitney Test showed a statistically significant difference (p &lt; 0.001) in E values of the two groups in the second measurement, indicating that the two finishing techniques were chromatically different after exposure to the staining agent. This result seems to favor the Astropol technique, as shown in Table 3.

Table 3

## Discussion

This in vitro study aimed to compare two finishing and polishing techniques and assess their impact on restoration color stability over time. A secondary aim was to assess the durability of each protocol over time. The universal composite resin used adapts to the color of surrounding dental tissue through structural color technology rather than conventional pigment additives. Despite its innovative optical properties, recent literature underscores the need for further research on this universal resin-based restorative material. The present findings should be interpreted in light of this restoration-centered experimental model. Previous studies using standardized composite specimens have provided important information on the intrinsic optical behavior of universal composites, including pigment penetration, bleaching response, and the influence of surface finishing on material permeability. However, single-shade composites are specifically designed to achieve aesthetic integration through interaction with surrounding tooth structure. For this reason, their performance cannot be fully assessed using flat composite specimens alone. By using standardized Class V cavities on extracted molars, the present study incorporated anatomical substrate, restoration margins, adhesive interface, and adjacent sound dental tissue into the color evaluation. This design allowed the comparison between Astropol and Enhance to be interpreted not only as a comparison between polishing systems, but also as a test of whether a simplified finishing strategy is sufficient to preserve the color-blending behavior of Omnichroma over time. The greater discoloration observed after staining in the Enhance group suggests that procedural simplification may have limits and that the long-term aesthetic performance of single-shade composites remains dependent on the quality of the final polished surface. Differences in performance between the Astropol and Enhance systems, particularly regarding long-term color stability, can be critically linked to their distinct abrasive mechanisms and resulting surface topography. The multi-step Astropol system, with its sequential use of progressively finer abrasive particles (Astropol F, P, HP), is designed to achieve a highly smooth and glossy surface (reported Ra values of 0.0549 m). Such a refined surface minimizes the retention of staining agents, thereby enhancing color stability over time. By contrast, the Enhance system, primarily a finishing tool, was used as a standalone 'one-step' approach in this study, likely resulting in a comparatively rougher surface (reported Ra values of 0.4631 m). Increased surface roughness may facilitate extrinsic stain accumulation, leading to higher E values in the Enhance group after immersion in the staining solution. As specified in the Materials and Methods section, the Enhance instrument was intentionally used as a standalone one-step approach in this study. This choice was made to assess the effect of a simplified finishing protocol on color stability, rather than to evaluate the complete manufacturer-recommended Enhance/PoGo sequence. Therefore, the greater discoloration observed in the Enhance group should not be interpreted as evidence of inferior performance of the full Enhance/PoGo system, but rather as an indication that omitting a dedicated final polishing step may reduce long-term chromatic stability. This study investigated whether finishing and polishing procedures influence the color blending effect and its preservation over time. An in vitro approach was chosen to ensure standardized conditions, enabling consistent and reproducible assessment of the composite's physical properties, which are difficult to obtain in vivo. Spectrophotometric analysis was used to standardize color measurements and ensure uniformity among specimens. According to Chen et al. (2012), spectrophotometers provide highly accurate and precise color-matching results despite their predominant use in anterior restorations (12). Several factors may influence the color stability of composite materials, including organic matrix type, polymerization degree, and oral hygiene conditions. Surface roughness plays a crucial role in discoloration susceptibility. Beltrami et al. demonstrated that reducing filler particle size minimizes discoloration, whereas Pal's research reported that a smoother surface prevents plaque accumulation and improve restoration durability and aesthetic quality. Aydin further observed that smaller resin matrix particles enhance finishing and polishing effectiveness and reduce both surface roughness and discoloration (13 - 18). A systematic review comparing the Astropol (Ivoclar Vivadent Group, Liechtenstein) and Enhance (Dentsply Sirona, Milford, Delaware, USA) reported Ra values of 0.0549 m and 0.4631 m, respectively (19). Astropol demonstrated superior smoothness, whereas Enhance was favored for efficiency. In clinical practice, reduced treatment time and lower costs frequently favor the use of one-step systems, although this simplification may affect restoration quality. The findings of the present study indicate that although both systems initially demonstrated acceptable color performance, the Astropol protocol maintained superior color stability following prolonged exposure to staining agents. Further research is needed to determine whether Astropol's efficacy extends across different composite resins or if material-specific interactions influence outcomes. Additionally, filler characteristics impact not only polishability but also wear resistance, suggesting a need to evaluate multiple composite types over time under finishing and polishing conditions. Notably, although the Enhance technique produced higher Ra values, its short-term clinical performance remained satisfactory. The use standardized Class V cavities on extracted molars, rather than composite resin discs, was driven by the aim to enhance the clinical relevance and ecological validity of the study. Although composite discs offer highly controlled and reproducible surfaces for color measurements, they do not fully replicate anatomical complexity, adhesive interface, and optical interaction present in clinical restoration. Class V cavities, positioned on the buccal surface, enabled evaluation of color blending and stability within a more realistic setting, including challenges associated with marginal adaptation and light reflection from natural tooth tissues. Although thermocycling is widely used to simulate thermal aging and oral temperature fluctuations in in-vitro studies, it was intentionally excluded from the present investigation. This choice was made to isolate the effect of the finishing/polishing procedures on color stability after staining exposure. Nevertheless, the absence of thermocycling represents a relevant limitation, as thermal changes may affect the resin matrix, filler-matrix interface, water sorption, marginal integrity, and stain susceptibility of composite restorations. Future studies should incorporate thermocycling and mechanical aging to better simulate the combined thermal and functional stresses occurring in the oral cavity. A significant limitation of spectrophotometric studies involving the E parameter is the absence of a universally accepted perceptibility threshold. While some studies set this threshold at E = 3.0 or 2.7, others suggest values below 1 are imperceptible to the human eye and that values between 1 and 2 discernible only by trained observers (20 - 24). This study adopted a E threshold of 2, acknowledging that all measurements exceeded this value with considerable variability, likely influenced by initial tooth color and molar characteristics. Previous literature, such as AlHabdan et al. (2022), reported substantial color discrepancies in anterior restorations (E = 6.474), findings that are consistent with the present results (2 , 25 , 26). However, Bompolaki suggested that single-shade composites, may be better suited for small restorations and monochromatic teeth despite simplifying shade selection procedures. Another limitation concerns cavity size (4 mm mesiodistally and in depth), which may restrict color blending in posterior restorations. One strength of this study is the prolonged exposure to staining agents, simulating over a year of clinical conditions (11 , 19). Future research should incorporate intermediate measurements to track progressive discoloration over time. Additionally, evaluating intermediate polishing methods may help establish maintenance protocols for long-term restoration aesthetics. While a systematic review suggests that combining different polishing techniques does not significantly affect surface roughness, its influence on color blending remains unclear. Exploring the potential benefits of a synergistic approach over time could provide further insights into optimizing restorative outcomes. It is important to acknowledge that the absence of a statistically significant difference (e.g., p &gt; 0.05) does not necessarily imply clinical equivalence or comparability between groups. A non-significant result indicates insufficient evidence to reject the null hypothesis of no difference, but it does not confirm the absence of a true difference, especially without conducting specific non-inferiority or equivalence trials designed for such conclusions. Therefore, although the initial color performance of the two techniques did not statistically differ, drawing definitive conclusions about their absolute comparability in the immediate post-polishing phase should be done cautiously. A significant limitation of this study is the use of a single shade composite resin. Only one single-shade composite resin, Omnichroma, was evaluated. This allowed a focused assessment of a structural-color composite with specific optical behavior; however, the results cannot be generalized to other resin-based composites with different monomer composition, filler size, filler loading, translucency, or color-adjustment potential. Comparative studies including different single-shade and conventional multi-shade composites are needed to determine whether the observed influence of finishing and polishing protocols is material-dependent. Future research should investigate the chromatic performance of various composite resins under similar finishing and polishing protocols to provide a broader understanding of material-specific interactions. A further limitation is that the Enhance protocol evaluated in this study was a simplified standalone one-step approach and did not correspond to the complete manufacturer-recommended Enhance/PoGo polishing sequence. Consequently, the findings should be interpreted specifically in relation to the experimental protocol tested and should not be generalized to the complete system.

## Conclusions

The multi-step Astropol finishing and polishing protocol appeared more effective in maintaining the color stability of Omnichroma restorations than the simplified standalone one-step Enhance protocol tested in this study. However, these findings should not be generalized to the complete manufacturer-recommended Enhance/PoGo polishing sequence, which includes an additional polishing step not evaluated in the present investigation.

## Figures and Tables

**Table 1:** ΔE values 24 hours after finishing/polishing and after 14 days of immersion in the staining agent for group 1.

Group 1 (Astropol)	T1 ΔE AB	T2 ΔE AB
A1	3.34	3.55
A2	3.29	3.56
A3	3.74	3.74
A4	3.95	4.46
A5	3.8	5.48
A6	3.91	5.07
A7	5.06	5.86
A8	3.51	2.36
A9	3.3	5.35
A10	3.78	6.23
A11	5.67	4.59
A12	3.17	4.2
A13	3.21	3.68
A14	2.85	3.27
A15	3.86	4.86
A16	2.51	3.29
A17	13.76	15.94
A18	4.11	5.25
A19	4.2	4.74
A20	3.73	3.84
A21	6.96	7.45
A22	5.27	5.49
A23	3.95	4.15
A24	3.85	4.56
A25	5.73	5.85
A26	4.67	6.25
A27	5.65	7.63
A28	2.71	3.37
A29	2.87	3.05
A30	6.63	8.03

1

**Table 2:** ΔE values 24 hours after finishing/polishing and after 14 days of immersion in the staining agent for group 2.

Group 2 (Enhance)	T1 ΔE AB	T2 ΔE AB
E1	3.76	6.69
E2	4.67	8.49
E3	3.5	6.08
E4	5.69	8.31
E5	4.25	7.43
E6	6.67	8
E7	4.8	7.43
E8	4.05	7.64
E9	6.58	8.21
E10	5.27	7.68
E11	3.68	6.76
E12	3.45	5.21
E13	6.07	6.44
E14	4.71	10.18
E15	3.09	6.72
E16	3.64	8.66
E17	5.55	8.85
E18	4.94	9.63
E19	5.45	7.23
E20	2.27	7.24
E21	6.06	8.75
E22	3.66	10.25
E23	8.15	10.66
E24	4.34	8.1
E25	5.98	10.49
E26	6.84	9.01
E27	4.18	6.78
E28	3.8	6.78
E29	4.32	6.51
E30	4.18	7.84

2

**Table 3:** Descriptive statistics of ΔE values for Group 1 and Group 2.

		Mean value	SD
Group 1 (Astropol)	T1 ΔE AB	4.435	2.091
	T2 ΔE AB	5.171	2.466
Group 2 (Enhance)	T1 ΔE AB	4.787	1.307
	T2 ΔE AB	7.935	1.375

3
